# Family physicians' information seeking behaviors: A survey comparison with other specialties

**DOI:** 10.1186/1472-6947-5-9

**Published:** 2005-03-22

**Authors:** Nancy L Bennett, Linda L Casebeer, Robert Kristofco, Blanche C Collins

**Affiliations:** 1 Department of Continuing Education, Harvard Medical School, Boston, Massachusetts, USA; 2 Division of Continuing Medical Education, University of Alabama, Birmingham, Alabama, USA; 3 School of Public Health, University of Wisconsin at Lacrosse, USA

## Abstract

**Background:**

Using technology to access clinical information has become a critical skill for family physicians. The aims of this study were to assess the way family physicians use the Internet to look for clinical information and how their patterns vary from those of specialists. Further, we sought a better understanding of how family physicians used just-in-time information in clinical practice.

**Methods:**

A fax survey was provided with 17 items. The survey instrument, adapted from two previous studies, was sent to community-based physicians. The questions measured frequency of use and importance of the Internet, palm computers, Internet CME, and email for information seeking and CME. Barriers to use were explored. Demographic data was gathered concerning gender, years since medical school graduation, practice location, practice type, and practice specialty.

**Results:**

Family physicians found the Internet to be useful and important as an information source. They were more likely to search for patient oriented material than were specialists who more often searched literature, journals and corresponded with colleagues. Hand held computers were used by almost half of family physicians.

**Conclusion:**

Family physicians consider the Internet important to the practice of medicine, and the majority use it regularly. Their searches differ from colleagues in other specialties with a focus on direct patient care questions. Almost half of family physicians use hand held computers, most often for drug reference.

## Background

As an information-intensive specialty without patient limits of age, gender, or medical presentation, family physicians require a number of different resources to cover the broad scope of practice. A critical clinical skill for family physicians is timely access to that wide variety of clinical information sources that contribute to patient care decisions. Specific questions about patient management arise in daily practice (about 3.2 questions for every 10 patients seen), with drug-prescribing queries being the most common type of question [1]. Pursuing answers to questions that arise only occurs about a third of the time [1]. The most frequent motivation to track questions comes from the belief that a definitive answer exists or the patient's problem is urgent [2]. It is estimated that about half of questions may readily be answered by information in a clinical record, one-quarter of questions require traditional resources as journals or textbooks, and one-quarter of the questions require synthesis of information about a specific patient with a biomedical knowledge base [3].

Essential to the use of any information source is the probability of success in quickly and accurately finding the desired information. For the questions they pursued, family physicians spent an average of less than 2 minutes finding an answer using traditional textbooks and journals [1]. compared to a study of a palmtop drug reference system where it took a group of physicians only 20 seconds to find answers to their questions [4]. Technologies improve the quality of care family physicians provide by improving access to necessary information. Care may be fragmented or diminished and less evidence based when access is not readily available or available only through specialists [5,6].

As a global information source, the Internet provides extensive options to search for answers, and may influence the way family physicians shape their questions and look for responses. Its importance in clinical practice has been documented [5,7-9]. Many physicians have also adopted the use of handheld computers for reference materials and to access necessary information at the point of care [10]. One study indicates that Personal Digital Assistants (PDAs) were used in 64% of outpatient clinical facilities, with 69% of PDA users accessing pharmaceutical information [11].

One approach to better understanding family physicians' information needs and how they manage information is to look at how they use resources on the Internet and hand held devices to access information. This study focuses on the ways that family physicians use the Internet to look for information in their practice, and how their information seeking patterns vary from those of specialists. We hypothesized that family physicians would seek a broader array of information resources that were directly linked to patient care when compared to specialists. Further, we wanted to understand more about just-in-time information for use in clinical practice.

## Methods

Patterns of current Internet use by family physicians and specialists were assessed and compared using a survey instrument with 17 items. The survey was adapted from two previous studies [8,9]. Three items measured frequency of use of the Internet, palm computers, Internet CME, and email. Four items measured type of use, including clinical information and specific patient problems, for the Internet, palm computers, and email. In four items, physicians were asked to rank the importance of the Internet for information seeking and CME. Using a Likert scale, three items were used to determine physician beliefs about the Internet. Three additional items measured barriers, type of resources available for searching, and choice of potential CME courses. Demographic data was gathered concerning gender, years since medical school graduation, practice location, practice type, and practice specialty. The survey was sent by facsimile transmission (Fax) during the period of December, 2002-January, 2003. The fax broadcast method of surveying effectively elicits responses from community-based practicing physicians [12], and avoids the bias of surveying only those physicians currently using the Internet. Previous studies have indicated that virtually all physicians in the United States have access to the Internet [8,9,11].

The population of interest for this survey was defined as U.S. physicians of all specialties in active practice, according to the most current American Medical Association physician listing; 518,000 physicians were identified with 69,000 family physicians. A power calculation determined that a sample size of 2200 was needed to generalize to the total population of U.S. physicians in terms of age and gender. Cochran's sampling technique was used to determine the power required for the study, with a margin of error of 5%, and 95% confidence [13]. In addition, the demographic characteristics of the sample of 2200 were compared to the demographic characteristics of the overall group of 518,000 and tested for differences to further assess the representativeness of the sample.

Surveys were faxed to a random sample stratified to include all major specialties drawn from the overall pool of U.S. physicians. Responses were solicited until a usable sample of 2200 surveys had been received. Each survey was personalized with the individual physician's name and fax number before faxing. Directions for returning the survey by fax included an 800-fax number to a designated fax broadcaster. Each returned survey was scanned, and electronic copies were sent by email to the Division of Continuing Medical Education, University of Alabama School of Medicine for data entry.

Survey responses were entered into an ACCESS database for analysis. This study represents a subanalysis of data from the overall study, comparing responses of family physicians to those of specialists. Family physicians were classified based on self-identification. Frequency distributions and means were calculated for each survey item. Demographic items and survey items were cross-tabulated and analyzed using Chi-square analysis.

## Results

A total of 2,200 usable responses required by the power calculation to generalize to the overall population of U.S. physicians were received by January 31, 2003; in the month following the solicitation of responses, an additional 194 usable responses were received and were included in the analysis. Of the 457 family physicians that responded, 72.7% were male and 27.4% female; 39.6% practiced in rural areas, and 44.8% reported graduating from medical school more than 20 years earlier. This demographic profile was compared to the American Medical Association profile of U.S. family physicians and no significant differences were found [14].

The majority (59%) of family physicians regularly use the Internet to access clinical information daily or weekly; they also regularly access the Internet for personal use and for email. Nearly half (47%) reported access by modem. Family physicians' responses differed from survey responses for other specialties in terms of strategies for seeking clinical information, as summarized in Table 1.

**Table 1:** Physicians' Internet Use

**Purpose**	**Family Medicine [%]**	**Specialist [%]**	**χ^2^**	**df**	**p-value**
Personal use	83.0	86.4	3.87	1	0.049
E-mail personal	78.7	81.7	2.16	1	.14
Literature searching	61.5	74.2	31.29	1	< .0001
Accessing online journals	57.6	67.0	14.40	1	.0001
Searching for patient-specific information	66.7	54.4	21.23	1	.0001
Professional association updates	47.3	48.6	0.32	1	.57
Consultation with colleagues	11.7	21.5	21.66	1	<.0001
Prescription/patient orders	6.0	3.4	5.85	1	.02

In comparing the importance of the Internet to other sources of clinical information, family physicians rated journals first, followed by local and national CME meetings, and then websites. However, the majority (73%) believed the Internet was useful and important to physicians.

More than half (54%) of family physicians reported confidence in using the Internet to find medical information; however, 14% were not at all confident. Family physicians were more likely to search for information related to a patient problem while other specialists were more likely to search for the latest research on a specific topic (chi square = 10.26, df = 3, p = 0.01). When addressing a specific patient problem, family physicians were more likely to be seeking information on diagnosis/management (73%), patient education materials (58%), followed by guideline summaries (49%). Specialists were similar except for a difference in searching for patient education materials (37%, chi square = 64.54, df = 1, p = < 0.0001).

Credibility was ranked as the most important characteristic of the Internet related to clinical information by both family physicians and physicians in other specialties. Family physicians were more likely than other specialists to cite "too much information to scan" as a barrier to clinical information seeking (p =.0004) on the Internet. Other barriers are summarized in Table 2.

**Table 2:** Physician Internet Barriers

**Barrier**	**Family Medicine [%]**	**Specialist [%]**	**χ^2^**	**df**	**p-value**
Too much information to scan	58.6	48.9	12.53	1	.0004
Specific information not available	44.0	48.3	3.40	1	.065
Navigation/searching difficulties	61.3	60.4	0.09	1	.77
Too slow	32.9	28.6	2.83	1	.09
Software incompatibilities	18.3	21.2	1.83	1	.176
Downloading information too difficult	30.6	29.6	0.25	1	.62

Family physicians were also concerned with the relevance of clinical information. They were more likely than specialists to use handheld computers (p =.0004) with nearly half (49%) of family physicians reporting use, most frequently for drug reference. Table 3 summarizes handheld computer usage.

**Table 3:** Hand Held Computer Functions

**Identified Useful Function**	**Family Medicine [%]**	**Specialist [%]**	**χ^2^**	**df**	**p-value**
Drug References	94.1	80.3	23.48	1	< .0001
Clinical Guidelines	54.5	45.0	6.27	1	.01
E-mail	5.9	13.2	8.84	1	.003
Patient specific information	20.7	19.3	0.10	1	.75
Web search	5.0	6.9	1.21	1	.27

## Discussion

Because of the broad scope of family practice and the exponential increase in medical knowledge, mastery of technology is a core skill for family practice. This study looked at the ways in which family physicians make use of technology, and how their information seeking behavior compares to colleagues in other specialties. Family physicians consider the Internet important to the practice of medicine. The majority report regular use as a source of clinical information, driven by clinical questions that arise during the care of specific patients rather than looking for new research findings. This study indicates they differ from colleagues in other specialties in the kinds of clinical information needs that lead to a search for information.

Family physicians search for specific material that will benefit a patient or group of patients, often via handheld computers. The use of handheld computers points to interesting questions about whether there is greater use of just-in-time information by family physicians. That is consistent with the findings of family physician use of handheld computers in outpatient settings [11]. Understanding current best practices and clinical guidelines in order to respond to individual variation and needs among patients is supported by access to technology. Specialists, on the other hand, require in-depth knowledge in a relatively narrow area, with a need to use technology for access to cutting edge research and journals, and contact with a more limited population of colleagues, many of whom may be at a distance. Both groups found problems with navigation and lack of speed in searching.

A number of obstacles to using evidence to answer physicians' patient care questions have been identified. This study confirms Internet searching difficulties for both specialists and family physicians, with an extensive amount of information to scan, and lack of specificity for available information. These obstacles appear to be greater for family physicians in their quest for patient-specific information. Specialists find less difficulty in their primary information targets of literature searching, online journal use, and consultation with colleagues via email. As medical information expands and the number of web pages increases, these barriers will grow in the years to come.

When family physicians search for clinical information, the Internet may be accessed by desktop/laptop or hand-held computers. Handheld devices allow immediate access to answers to many of the clinical questions family physicians ask, especially at the point of care. Family physicians were significantly more likely than physicians of other specialties to use hand-held computers, and more likely to use them for drug references and clinical practice guidelines. This finding is consistent with the findings for other primary care physicians. A recent survey of internists found that 80% of hand held computer owners used them to access drug information [16]. Handheld computers may also be used to access electronic medical textbooks, downloadable journals, medical calculators, patient-tracking programs, billing and coding software, word processing and utility software, and web access and content, with future possibilities of dictation of clinical notes and email [17]. As more physicians integrate hand-held computers into their practices, the number and quality of clinical applications will continue to grow.

Gaps in knowledge related to drug prescribing is one of the most common causes of serious medication errors [18]. A recent study of the clinical use of a hand held drug reference guide demonstrated that physicians felt this technology saved time during information retrieval, was easily incorporated into their workflow, and that it reduced the rate of preventable adverse drug events [4]. The use of hand held computers for referencing clinical practice guidelines and drug questions by half of the family physicians surveyed indicates that hand held computers are becoming more rapidly integrated into the clinical encounter and provide one step in addressing patient safety issues.

## Conclusion

Family physicians deal with the broadest of clinical knowledge bases, yet two-thirds of their clinical questions go unanswered [1]. They are most likely to pursue those questions with a high probability of finding an answer or with an urgent clinical application [2]. Technology is one tool to respond to these problems. Family physicians have access to technology for information and are using it. They consider the Internet an important information source, and are confident in their ability to search for information. Compared to specialists, family physicians direct more attention to patient care questions, perhaps at the point of care. However, when they use the Internet for clinical information, family physicians can be overwhelmed by the amount of clinical information, their inadequate searching skills and their lack of confidence that they will be able to answer a question. The increased use of handheld computers points to more potential use at the point of care clinical encounter; they appear to be particularly useful in accessing drug information and clinical practice guidelines and likely will grow in numbers of users and types of applications. The use of handheld computers may contribute to an effort to increase patient safety. Although technology offers access to information, it also offers a series of challenges to medical educators and researchers, and to those who design technology applications to create bridges for practitioners to the information they need to practice medicine.

## Competing interests

The author(s) declare that they have no competing interests.

## Authors' contributions

NB, LC, RK developed the survey form and study protocol. BC contributed to literature, and supervised administration of the survey. All authors contributed to the writing of the article, and read and approved the final manuscript.

## Pre-publication history

The pre-publication history for this paper can be accessed here:


